# Structure, Expression, and Functional Analysis of the Hexokinase Gene Family in Cassava

**DOI:** 10.3390/ijms18051041

**Published:** 2017-05-12

**Authors:** Meng-Ting Geng, Yuan Yao, Yun-Lin Wang, Xiao-Hui Wu, Chong Sun, Rui-Mei Li, Shao-Ping Fu, Rui-Jun Duan, Jiao Liu, Xin-Wen Hu, Jian-Chun Guo

**Affiliations:** 1 College of Agriculture, Hainan University, Haikou 570228, China; mengtinggeng8908@163.com (M.-T.G.); 13876830997@163.com (Y.-L.W.); 2 Key Laboratory of Biology and Genetic Resources of Tropical Crops, Ministry of Agriculture, Institute of Tropical Bioscience and Biotechnology, Chinese Academy of Tropical Agricultural Sciences, Haikou 571101, China; yaoyuan@itbb.org.cn (Y.Y.); liruimei@itbb.org.cn (R.-M.L.); fushaoping@itbb.org.cn (S.-P.F.); duanruijun@itbb.org.cn (R.-J.D.); liujiao@itbb.org.cn (J.L.); 3 Prisys Biotechnologies Company Limited, Shanghai 201203, China; xiaohui.wu@prisysbiotech.com; 4 College of Life Science and Biotechnology, Heilongjiang Bayi Agricultural University, Daqing 163319, China; sun780347812@163.com

**Keywords:** cassava, hexokinase, gene expression, yeast complementation, enzyme activities

## Abstract

Hexokinase (HXK) proteins play important roles in catalyzing hexose phosphorylation and sugar sensing and signaling. To investigate the roles of HXKs in cassava tuber root development, seven *HXK* genes (*MeHXK1–7*) were isolated and analyzed. A phylogenetic analysis revealed that the MeHXK family can be divided into five subfamilies of plant HXKs. MeHXKs were clearly divided into type A (MeHXK1) and type B (MeHXK2–7) based on their N-terminal sequences. MeHXK1–5 all had typical conserved regions and similar protein structures to the HXKs of other plants; while MeHXK6–7 lacked some of the conserved regions. An expression analysis of the *MeHXK* genes in cassava organs or tissues demonstrated that *MeHXK2* is the dominant HXK in all the examined tissues (leaves, stems, fruits, tuber phloems, and tuber xylems). Notably, the expression of *MeHXK2* and the enzymatic activity of HXK were higher at the initial and expanding tuber stages, and lower at the mature tuber stage. Furthermore, the HXK activity of MeHXK2 was identified by functional complementation of the HXK-deficient yeast strain YSH7.4-3C (*hxk1*, *hxk2*, *glk1*). The gene expression and enzymatic activity of MeHXK2 suggest that it might be the main enzyme for hexose phosphorylation during cassava tuber root development, which is involved in sucrose metabolism to regulate the accumulation of starch.

## 1. Introduction

In plants, sucrose is the primary end product of photosynthesis, which can be metabolized in source tissues (leaves), or exported out of the source tissues to sink tissues [1]. During sucrose metabolism, it firstly must be cleaved into hexoses (glucose and fructose) by either invertase (EC3.2.1.26) or sucrose synthase (EC 2.4.1.13) [2]. Then, the hexoses are phosphorylated by hexokinases (HXKs) or fructokinases and the phosphorylated hexoses become involved in metabolic processes [3]. HXKs phosphorylate glucose and fructose, while fructokinases specifically phosphorylate fructose [4,5]. Both of these enzyme families play important roles in the regulation of plant sugar metabolism and sugar signaling [1].

Plant HXKs are encoded by a gene family of about 3–10 members that have been found in many plant species [1]. The HXK family in *Arabidopsis thaliana* has six members, among which three proteins can phosphorylate hexoses, while the other three proteins lack this catalytic activity and are named as HXK-like (HKL) proteins [6]. Various isoforms of *HXK* genes have been found in the genomes of plants including *Oryza sativa*, *Zea mays*, *Populus trichocarpa*, *Nicotiana tabacum*, *Lycopersicon esculentum*, and *Vitis vinifera* [7]. The plant HXKs are classified into two groups (type A and type B) according to their N-terminal amino acid sequences [8]. Type-A HXKs have a chloroplast transit peptide and localize within plastids, such as AtHXK3 in *A. thaliana*, OsHXK4 in *O. sativa*, and LeHXK4 in *L. esculentum* [9]. Type-B HXKs share a common hydrophobic membrane anchor domain and are associated with mitochondria, such as AtHXK1–2 and AtHLK1–3 in *Arabidopsis*, OsHXK2, 3, 5, 6, 9, and 10 in *O. sativa*, and LeHXK1–3 in *L. esculentum* [9]. Recently, Cheng et al. (2011) identified that OsHXK1, 7, and 8 in *O. sativa* are located in the cytoplasm and nucleus, since they lack the plastidic transit peptide and the membrane anchor domain; thus, these enzymes are classified as type-C HXKs [10]. The variety and multiple subcellular localizations of HXKs indicate that these proteins may have functions in various physiological processes of plants, such as anther dehiscence, pollen germination [11], stomatal closure [12], germination [13], programmed cell death [14], and responses to abiotic or biotic stresses [15]. It has been identified that HXKs have important roles in sugar metabolism and sugar signaling [7,16]. AtHXK1 in *A. thaliana* and NtHXK1 in *N. tabacum* are dual-function enzymes that have hexose-phosphorylation activities and mediate sugar sensing [1].

HXKs are crucial elements of the sucrose metabolic pathway, which provides substrates for starch biosynthesis [17]. The transcript levels of *OsHXK7* and *OsHXK8* are high in rice seed during the starch-filling phase, while *AtHXK3* is most actively expressed in sink tissues (root and siliques) [18]. HXKs catalyze the phosphorylation of hexoses that are substrates in plastids for starch biosynthetic processes [19]. HXKs are involved in the transduction of sucrose-induced signals, which regulate the expression of genes involved in sucrose metabolism and starch biosynthesis [20,21]. For instance, HXK1 and HXK2 from *Vitis vinifera* play major roles in regulating the gene expression of cell wall invertases and sucrose synthases (*SuSy1* and *SuSy2*) during grape berry development. These enzymes are involved in sucrose phloem unloading and the control of carbohydrate accumulation in sink tissues [22]. The expression of the granule-bound starch synthase gene *OsGBSSII* from *O. sativa* is dependent on the sugar-regulating pathway of HXKs [23]. In the tubers of *Solanum tuberosum*, HXKs are involved in sugar-signaling pathways to modulate the translation and post-translational modification of ADP-glucose pyrophosphorylase, which adjusts the rate of starch synthesis [24].

Cassava (*Manihot esculenta* Crantz) is a tropical food crop that is a staple food for more than 700 million people in the tropical and subtropical regions of Asian and African countries [25]. The cassava tuber root is rich in starch (30% starch/g fresh weight) [26]. HXKs play a key role in cassava starch synthesis [19]. Cassava cultivar Fuxuan 01 (high tuber root starch) has a stronger ability to use the hexoses to synthesize starch than cultivar Huanan 124 (low tuber root starch) [27]. The expression of starch branching enzyme genes (*SBEI* and *SBEII*) in cassava tuber roots relies on abscisic acid signaling and sugar signaling, which is controlled by HXK activity [28]. However, the *HXK* genes of cassava have not been identified and cloned. A computational analysis using the cassava genome database showed that there are seven members of the *HXK* gene family in cassava (*MeHXK1–7*), and all of them were successfully cloned from a cassava cultivar (*M. esculenta* Crantz SC8). The evolutionary relationships, exon-intron structures, motif distributions, subcellular localizations, and three-dimensional (3D) structures of MeHXK proteins were investigated. The temporal and spatial expression of *MeHXK1–7* was analyzed by quantitative polymerase chain reaction (qPCR) in leaves, stems, tuber roots, flowers, and fruits. In addition, the differential expression and enzymatic activity of MeHXK1–7 were investigated in sink organs during tuber root development. Finally, the HXK activity of MeHXK2 was investigated through functional complementation experiments using the HXK-deficient yeast strain YSH7.4-3C (*hxk1*, *hxk2*, *glk1*). These results contribute to further understanding of the roles of HXK in sucrose metabolism in cassava tuber roots.

## 2. Results

### 2.1. Identification of MeHXK Family Members

**Figure 1:**
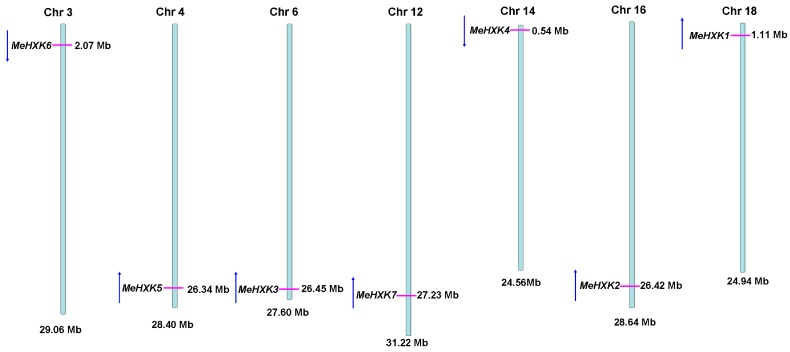
Chromosomal distribution of *MeHXK* genes in the cassava genome. Blue long lines represent the chromosome models, and the seven chromosomes are labeled. Pink short lines indicate the related position of each *MeHXK* gene in the cassava genome. Blue arrows indicate gene direction.

**Figure 2:**
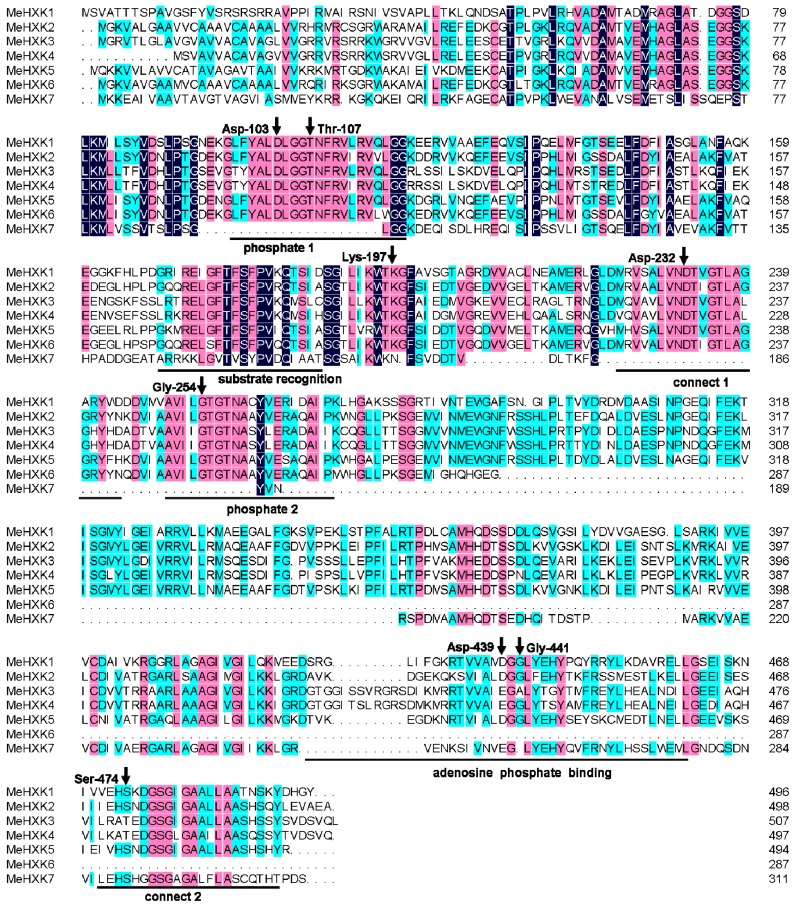
Amino acid alignment analysis of the identified cassava HXKs. Black lines indicate the regions with different functions. The black arrows show the eight conserved amino acids (counting the amino acid residues based on MeHXK1, the eight amino acids are Asp-103, Thr-107, Lys-197, Asp-232, Gly-254, Asp-349, Gly-441, and Ser-474), which likely correspond with the active residues of hexokinases (HXKs) proposed by Kuser et al. [29]. Dark-blue shading, pinkish shading and light blue shading reflect 100%, 75% and 50% amino acid residues conservation, respectively. The alignment was performed using DNAMAN 6.0 software (Lynnon LLC, San Ramon, CA, USA).

A BLAST analysis of the cassava genome database based on the *HXK* sequences of *Ricinus communis* and *A. thaliana* identified seven *HXK* genes (*MeHXK1–7*) in the cassava genome. The full-length cDNAs of *MeHXK1–7* were cloned by reverse transcription PCR. The cDNA sequences and the deduced amino acids of *MeHXK1–7* were deposited in GenBank under the following accession numbers: *MeHXK1* (KJ417433), *MeHXK2* (KJ417434), *MeHXK3* (KJ417435), *MeHXK4* (KJ417436), *MeHXK5* (KJ417437), *MeHXK6* (KJ417438), and *MeHXK7* (KJ417439). The open reading frames of *MeHXK1–7* varied in size from 861–1494 bp. MeHXK3 had the largest molecular mass (55,303.8 Da), while MeHXK6 had the smallest (30,714.7 Da). The theoretical isoelectric points of these HXKs were between 5.81–7.70 (Table 1). To investigate the genomic organization of *MeHXK1–7*, their positions on the cassava chromosomes were mapped. *MeHXK1–7* were found to be distributed among the seven cassava chromosomes chr 3, chr 4, chr 6, chr 12, chr 14, chr 16, and chr18 (Figure 1). Amino acid alignment analysis showed that *MeHXK1–7* share 35.92–89.74% identity (Figure 2). Most of these MeHXKs contain eight conserved residues; using numbering based on MeHXK1, the residues are Asp-103, Thr-107, Lys-197, Asp-232, Gly-254, Asp-349, Gly-441, and Ser-474. These conserved residues are involved in the HXK enzyme active site proposed in *Helianthus annuus*, *Citrus sinensis*, *Solanum lycopersicum*, *N. tabacum*, and *Saccharomyces cerevisiae* [29,30]. Homologous regions in MeHXKs were assigned according to the analysis of HXK2 from *S. cerevisiae* [30]. MeHXK6 lacks the C-terminal sequence that contains the conserved adenosine phosphate-binding and connects two regions. MeHXK7 lacks several sequences that contain the conserved regions of the phosphate 1, connect 1, and phosphate 2. A low-complexity region is shared among MeHXK3–4 as well as MeHXK1–2, 5, and 7 at the N-terminal side of the adenosine phosphate-binding region. MeHXK3–4 have an unusual insertion-deletion mutation at this region. MeHXK3–4 have most of the conserved phosphate 1, connect 1, phosphate 2, connect 2, and adenosine phosphate-binding regions (Figure 2).

**Figure 3:**
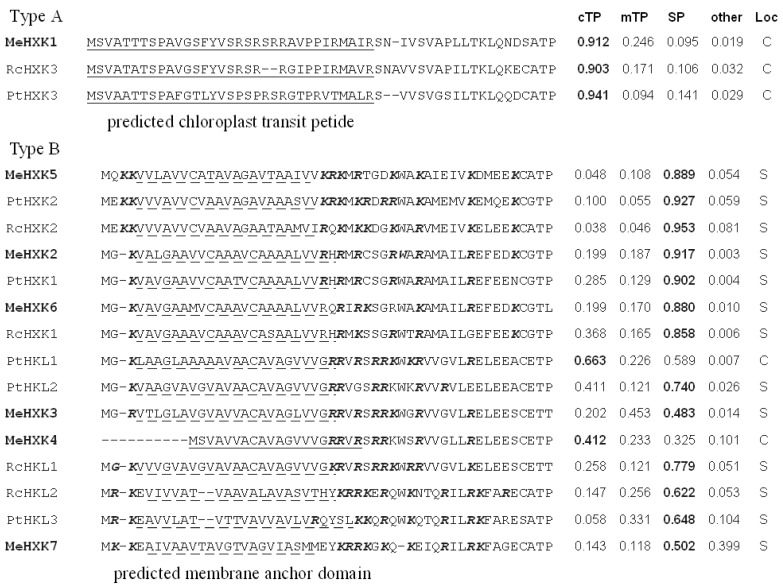
Subcellular localization prediction and N-terminal sequence analysis of the HXK proteins in *M. esculenta*, *R. communis*, and *A. thaliana*. The highest TargetP scores are written in bold font on the right side of Figure 3. cTP: chloroplast transit peptide score; mTP: mitochondrial targeting peptide score; SP: secretory pathway score; C: predicted chloroplast import; S: predicted secretory pathway. The black solid lines indicate predicted chloroplast transit peptides and the black dashed lines indicate predicted membrane anchor domains. Amino acid residues with a positive electrostatic potential located close to the predicted membrane anchor domain are shown in italics and bold.

The subcellular localizations of the MeHXKs were predicted using the TargetP 1.1 server (http://www.cbs.dtu.dk/services/TargetP/). Based on their N-terminal sequences, the HXKs from *M. esculenta* (MeHXK1–7), *R. communis* (RcHXK1–3 and RcHLK1–2), and *A. thaliana* (AtHXK1–3 and AtHLK1–2) were divided into two types, A and B (Figure 3). Among MeHXK1–7, only MeHXK1 has a putative 33 amino acid N-terminal cleavable chloroplast transit peptide, which is similar to that of the type-A HXKs RcHXK3 and AtHXK3 [6]. Thus, MeHXK1 may belong to a type-A plastid HXK. MeHXK2, 3, and 5–7 have a predicted N-terminal membrane anchor domain located near the positive electrostatic potential amino acid residues. This feature is similar to that of the type-B HXKs AtHXK1–2, AtHLK1–3, RcHXK1–2, and RcHLK1–2. Thus, these MeHXKs may be type-B membrane anchor HXKs, and might be associated with mitochondria, similar to HXKs previously identified in other plants. MeHXK4 has some characteristics of a type B HXK, but it does not contain an N-terminal membrane anchor domain; rather, it has a putative cleavable 20-amino-acid chloroplast transit peptide (Figure 3).

### 2.2. Structural Analysis of MeHXK1–7

**Figure 4:**
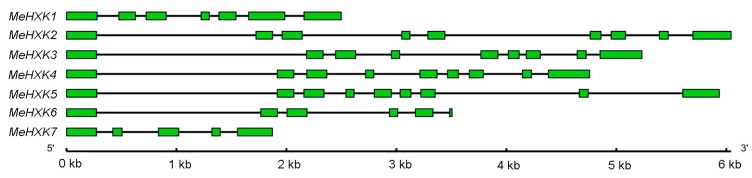
Exon-intron structure of *MeHXK1–7*. Introns are shown as black lines and exons are shown as green boxes.

The gene structures of *MeHXK1–7* were determined by aligning their cDNA sequences with the genomic sequence from the cassava genome database (http://www.phytozome.net/cassava). The results showed that *MeHXK1* has seven exons; *MeHXK2–5* have nine exons; *MeHXK6* has six exons and the last exon is smallest; and *MeHXK7* has five exons. The first exon structure is similar among *MeHXK1–7* (Figure 4).

### 2.3. Evolutionary Analysis of MeHXKs

To characterize the evolutionary relationships between the MeHXKs from cassava and other known HXKs from *R*. *communis* (RcHXK1–3 and RcHLK1–2), *P. trichocarpa* (PtHXK1–3 and PtHLK1–3), *Physcomitrella patens* (PpHXK1–11), *N. tabacum* (NtHXK1–6 and NtHLK1), *A. thaliana* (AtHXK1–3 and AtHLK1–2), *L. esculentum* (SlHXK1–4), and *O. sativa* (OsHXK1–10), an unrooted neighbor-joining tree was created using the MEGA7 program [31] based on their amino acid sequences.

**Figure 5:**
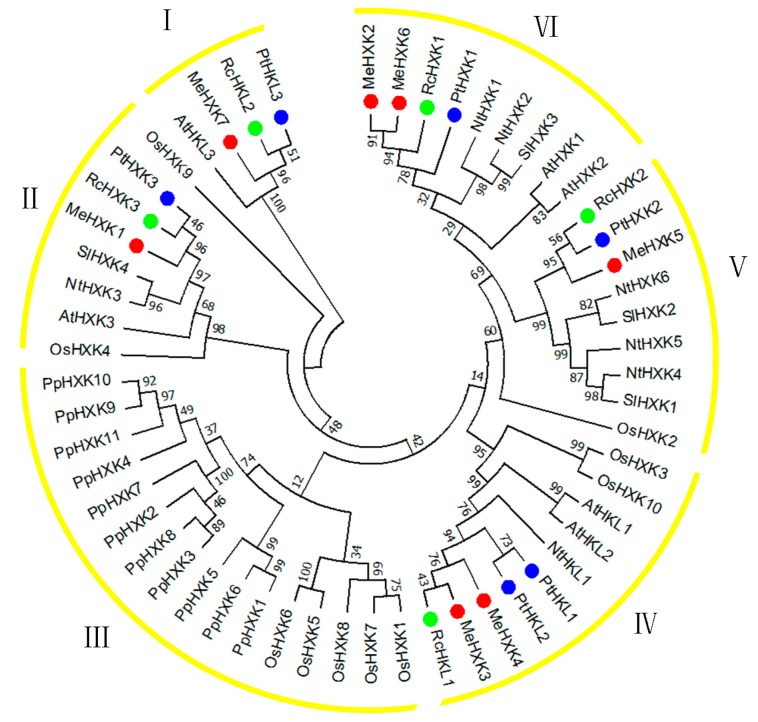
Phylogenetic analysis of HXK proteins from *Manihot esculenta*, *Ricinus communis*, *Populus trichocarpa*, *Nicotiana tabacum*, *Arabidopsis thaliana*, *Lycopersicon esculentum*, and *Oryza sativa*. HXK proteins were classified into six major sub-families (named as I, II, III, IV, V, and VI). The Neighbor-Joining tree was constructed using Molecular Evolutionary Genetics Analysis Version 7.0 (MEGA7). The values shown at the branch nodes are the confidence levels from 1000 replicate bootstrap samplings. Red dot indicate HXKs from *M. esculenta*. Blue dot indicate HXKs from *P. trichocarpa*. Green dot indicate HXKs from *R. communis*.

All 56 of these HXK proteins were classified into six major sub-families (groups I–VI) (Figure 5). Seven MeHXKs could be assigned to five subfamilies (groups I, II, and IV–VI) and had close relationships with their orthologous HXKs from *R. communis* and *P. trichocarpa*, which belong to the order Malpighiales. In group II, MeHXK1 shared 82.70% amino acid identity with the type-A HXKs RcHXK3 and PtHXK3. MeHXK7, RcHKL2, and PtHKL3 were grouped into group I and shared 66.80% amino acid identity. MeHXK3–4, RcHKL1, and PtHKL1–2 formed a clade in group IV and shared 91.2% amino acid identity. MeHXK5, RcHXK2, and PtHXK2 formed a clade in group V and shared 90.96% amino acid identity. MeHXK2, 6, RcHXK1, and PtHXK1 were grouped into group VI and shared 82.78% amino acid identity. All the HXKs from *M. esculenta*, *R. communis*, and *P. trichocarpa* were classified into groups I and IV–VII, which comprise type-B HXKs.

### 2.4. Motif Distribution in MeHXKs

**Figure 6:**
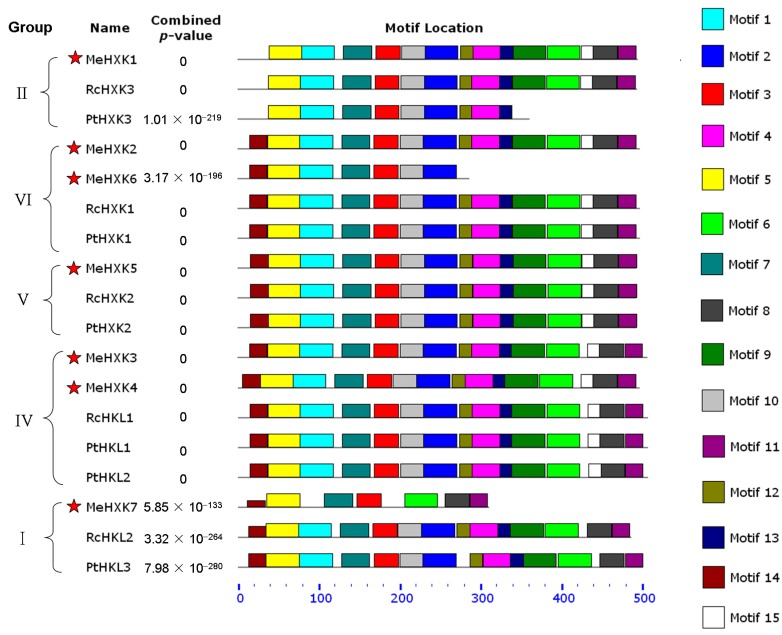
Motif distribution in HXKs from *M. esculenta*, *R. communis*, and *P. trichocarpa*. Motifs were analyzed using the MEME web server (https://www.swissmodel.expasy.org/). The motifs are represented by different colors. Red stars indicate HXKs from *M. esculenta*.

To further reveal the diversification of MeHXKs, the conserved motifs were predicted using the MEME web server (http://meme-suite.org/tools/meme) in comparison with the PtHXKs from *P. trichocarpa* and RcHXKs from *R. communis*. Fifteen motif sequences were identified in the tested HXKs (Figure 6). Based on the classifications from the phylogenetic analysis, the 18 HXKs from these species were categorized into five classes. Motifs 3, 5, and 7 were found in all 18 tested HXKs, while motif 14 was specifically distributed in the evolutionary groups I and IV–VII. Most HXKs in groups VI, V, and IV had all 15 motifs; while only seven motifs were distributed at the N-terminus of MeHXK6, and motifs 4, 6, 8, 9, 11–13, and 15 at the C-terminus were lost. In group I, motif 15 at the C-terminus of MeHXK7, RcHKL2, and PtHKL3 was lost; and MeHXK7 only had seven N-terminus motifs, while the eight C-terminus motifs were all lost. The results indicate that MeHXK1–5 all have a typical HXK motif distribution, while MeHXK6–7 lost several motifs during their evolution.

### 2.5. 3D Structures of MeHXKs

**Figure 7:**
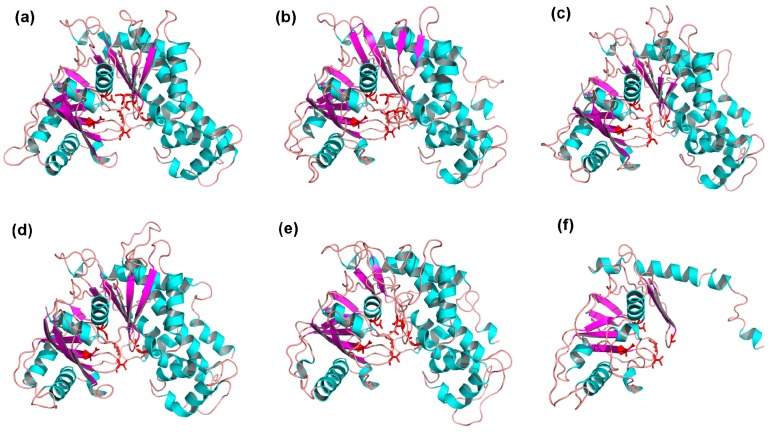
The cartoon representation of the predicted 3-dimensional structural models of MeHXK1–6. (**a**) MeHXK1; (**b**) MeHXK2; (**c**) MeHXK3; (**d**) MeHXK4; (**e**) MeHXK5; and (**f**) MeHXK6. Green structures represent α-helices, purple arrows indicate β-folds, lines represent random coils, and red stick structures represent the catalytic residues. The image was generated using the PyMOL program (Schrödinger, Inc., New York, NY, USA).

To obtain a reasonable theoretical structure of MeHXKs, protein homology modeling was performed using the HXK protein from *Schistosoma mansoni* (Protein databank ID 1BDG) as a template, which shared 40.82%, 39.91%, 39.62%, 39.20%, 42.32%, and 43.04% sequence identity with MeHXK1–6, respectively. The 3D structure of MeHXK7 was not constructed by homology modeling. The predicted 3D models of MeHXK1–6 were validated using the QMEAN server (http://swissmodel.expasy.org/qmean/cgi/index.cgi) for model quality estimation. The total QMEAN scores (the estimated model reliability between 0 and 1) of the predicted 3D models for MeHXK1–6 were 0.689 (Z-score: −0.93), 0.629 (Z-score: −1.65), 0.611 (Z-score: −1.91), 0.693 (Z-score: −0.92), 0.598 (Z-score: −2.03), and 0.474 (Z-score: −3.16), respectively. These results indicated that all the sequences of MeHXK1–6 matched the homologous templates well on the server, demonstrating that the models were reliable. MeHXK1–5 had very similar structural models. The sequences of the MeHXK proteins were distinctly folded into two domains. The N-terminus sequence formed a small domain, and the C-terminus sequence formed a large domain. These sequences were separated by a deep cleft that contains the residues involved in the enzyme’s active site. β-Sheets were located in the small domain or near the deep cleft. MeHXK6 only had the small domain because it lacked the C-terminal sequence (Figure 7).

**Figure 8:**
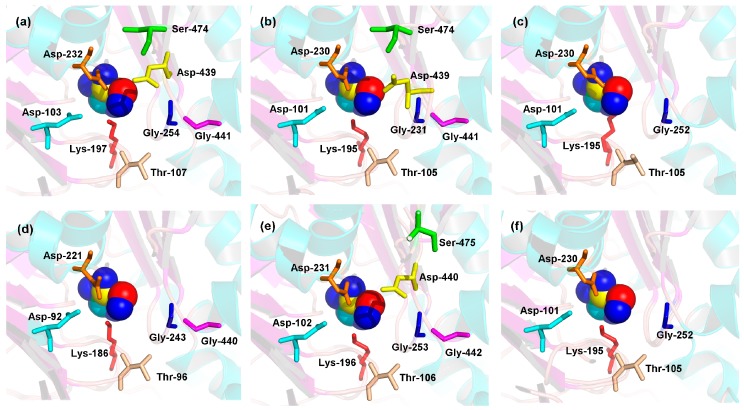
Distribution of the residues in the enzyme active sites of MeHXK1–6. (**a**) MeHXK1; (**b**) MeHXK2; (**c**) MeHXK3; (**d**) MeHXK4; (**e**) MeHXK5; and (**f**) MeHXK6. Spherical structures indicate glucose molecules and the colored stick structures represent the catalytic residues. The image was generated using the PyMOL program.

To predict the theoretical position of glucose when binding with the enzyme active sites of MeHXK1–6, the primary models of MeHXK1–6 were further structurally aligned with a model of the HXK protein from *S. mansoni* complexed with a glucose molecule (Protein databank ID 1BDG) using the PyMOL program (Schrödinger, New York, NY, USA). The results showed that the residues in the enzyme active site were located within the deep cleft and had a similar orientation with the glucose molecule (Figure 8). The enzyme active sites of MeHXK1–2 and 5 contained all eight of the conserved residues (using the numbering based on MeHXK1, the residues are Asp-103, Thr-107, Lys-197, Asp-232, Gly-254, Asp-439, Gly-441, and Ser-474). However, some of the residues in the enzyme active sites of MeHXK3, 4, and 6 were lost, with three residues (Ser-474, Asp-439, and Gly-441) missing from the enzyme active sites of MeHXK3 and 6. Finally, two residues (Ser-474 and Asp-439) were lost from MeHXK4.

### 2.6. Differential Expression Analysis of MeHXK1–7 in Cassava Organs or Tissues

**Figure 9:**
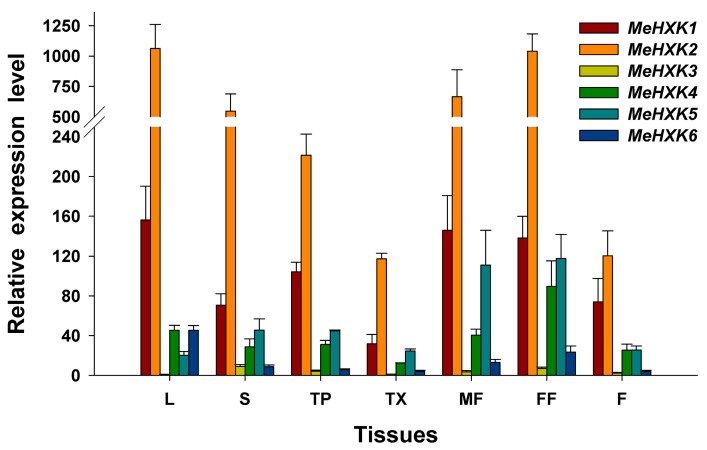
The differential expression of the *MeHXK* genes in cassava organs or tissues. The amount of *MeHXK* mRNA was normalized by *β-tubulin* mRNA. The expression of *MeHXK3* in leaves was used as a calibrator to compare with other genes for map-making. Each value is the mean ± SE of three biological replicates (*n* = 3). Notes: L, Leaf, S, Stem, TP, Tuber phloem, TX, Tuber xylem, MF, Male flower, FF, Female flower, F, Fruits.

The expression of *MeHXK* genes in leaves, stems, tuber phloem, tuber xylem, male and female flowers, and fruits was examined using quantitative polymerase chain reaction (qPCR). The result showed that the expression of *MeHXK2* was highest among all the *MeHXK* genes in all tested tissues. Its highest expression levels were found in leaves, stems, and flowers, where *MeHXK1* was the next most highly expressed *MeHXK* gene, but its expression was lowest in tuber xylem. The expression of *MeHXK4* was mainly in female flowers; that of *MeHXK5* was mainly in male and female flowers; and that of *MeHXK6* was mainly in leaves and female flowers. In the other tested organs and tissues, these genes had comparatively low expression levels. The expression of *MeHXK3* was low in all tested organs and tissues, and no expression of *MeHXK7* was detected (Figure 9).

### 2.7. Differential Expression of MeHXK Genes during Cassava Tuber Root Development

**Figure 10:**
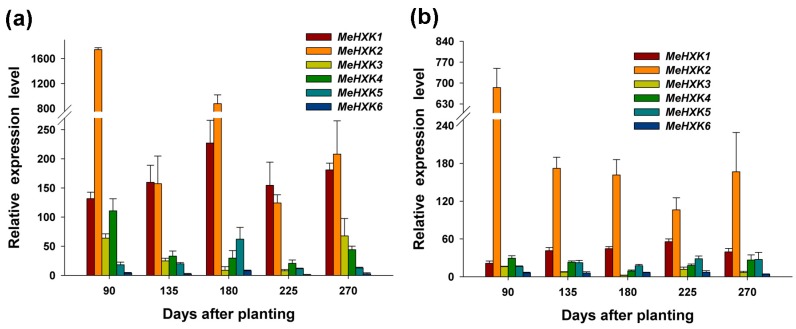
The differential expression analysis of *MeHXKs* in cassava tuber phloem (**a**) and tuber xylem (**b**) during tuber developmental stages. The differential expressions of *MeHXK* genes were examined using qPCR at the tuber initial stage (90 days), the tuber expanding stage (135 and 180 days), and the tuber maturity stage (225 and 270 days). Each value was the mean ± SE of three biological replicates (*n* = 3). The amount of *MeHXK* mRNA was normalized by *β-tubulin* mRNA. The expression of *MeHXK6* in tuber phloem at 225 days was used as a calibrator to compare with other genes for map-making.

The differential expression of *MeHXK* genes was examined in cassava tuber xylem and tuber phloem using qPCR at the initial tuber stage (90 days), expanding tuber stage (135 and 180 days), and mature tuber stage (225 and 270 days) during tuber root development. The expression of *MeHXK6* in tuber phloem at 225 days was used as a calibrator for comparison with other genes in tuber xylem and tuber phloem for map construction (Figure 10).

In tuber phloem, the expression of *MeHXK1* and *MeHXK2* was higher than that of the other *MeHXK* genes at all stages, and the highest expression of *MeHXK1* was at the initial tuber stage (90 days), followed by the later expanding stage (180 days), while the highest expression of *MeHXK1* was at the later expanding stage (180 days). *MeHXK3* was mainly expressed at the initial tuber stage (90 days) and the later mature tuber stage (270 days). *MeHXK4* was mainly expressed at the initial tuber stage (90 days) and *MeHXK5* was mainly expressed at the later expanding stage (180 days). *MeHXK6* maintained a low expression level at all stages.

In tuber xylem, *MeHXK2* maintained a high expression level at all tuber development stages, and its highest expression was at the tuber root initial stage (90 days). The other *MeHXK* genes maintained a low expression level at all stages.

### 2.8. The Activity of HXKs in Cassava Tubers during Tuber Root Development

**Figure 11:**
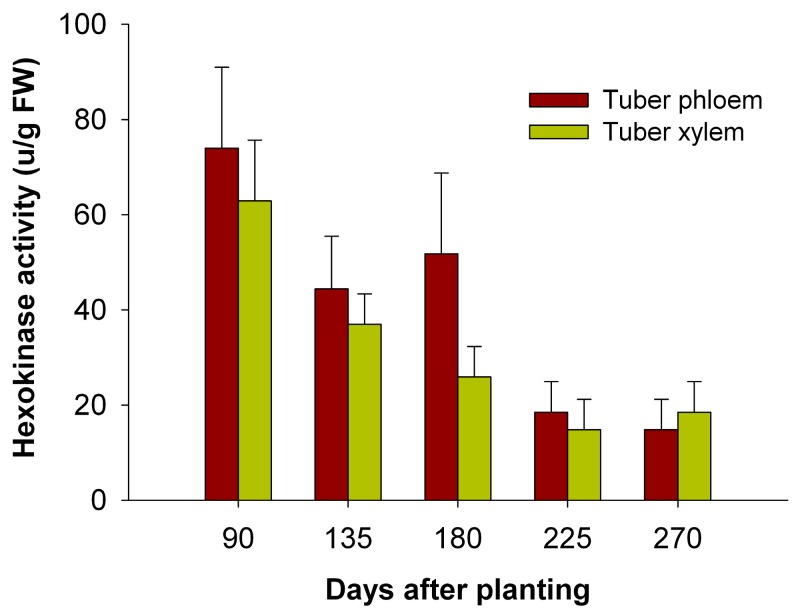
HXK activity profiles of cassava tuber phloem and xylem during tuber root development at the initial tuber stage (90 days), tuber expanding stage (135 and 180 days), and tuber maturity stage (225 and 270 days). Each value was the mean ± standard error of three biological replicates (*n* = 3).

To test the possible involvement of HXKs in sucrose metabolism at a sink organ during tuber root development, HXK activity was measured at the initial tuber stage (90 days), the expanding tuber stage (135 and 180 days), and the mature tuber stage (225 and 270 days). The results showed that HXK activity in the tuber phloem and xylem was highest at the initial tuber stage (90 days) and decreased during tuber root development (Figure 11).

### 2.9. Yeast Complementation of MeHXK2

**Figure 12:**
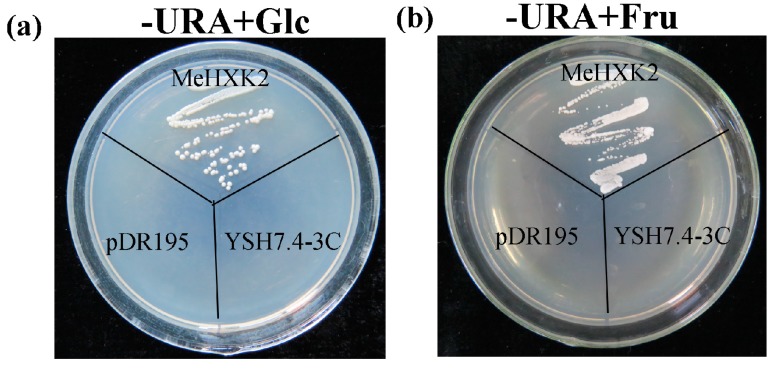
Complementation of the HXK-deficient yeast triple mutant YSH7.4-3C (*hxk1*, *hxk2*, *glk1*) with *MeHXK2*. −URA + Glc: the medium contained d-glucose as the sole carbon source and lacked uracil; −URA + Fru: the medium contained d-fructose as the sole carbon source and lacked uracil; MeHXK2: the pDR195-*MeHXK2* vector was transformed into YSH7.4-3C; PRD195: the empty pDR195 vector was transformed into YSH7.4-3C; YSH7.4-3C: the mutant yeast cells without any vector transformation.

*MeHXK2* was the most highly expressed MeHXK gene in cassava organs and tissues at multiple stages during tuber root development. Therefore, its activity was further examined in the yeast triple mutant YSH7.4-3C (*hxk1*, *hxk2*, *glk1*), which lacks endogenous HXK activity. This yeast mutant is unable to grow on either glucose or fructose as a sole carbon source. First, the cDNA of *MeHXK2* was inserted into a yeast expression vector pDR195 to generate pDR195-*MeHXK2*. The results showed that yeast cells transformed with the empty pDR195 vector could not grow on the selection medium containing glucose or fructose; whereas the yeast cells transformed with pDR195-*MeHXK2* could grow on these media (Figure 12). This result suggested that *MeHXK2* has a hexose phosphorylation function.

### 2.10. Kinetic Analysis of MeHXK2

**Figure 13:**
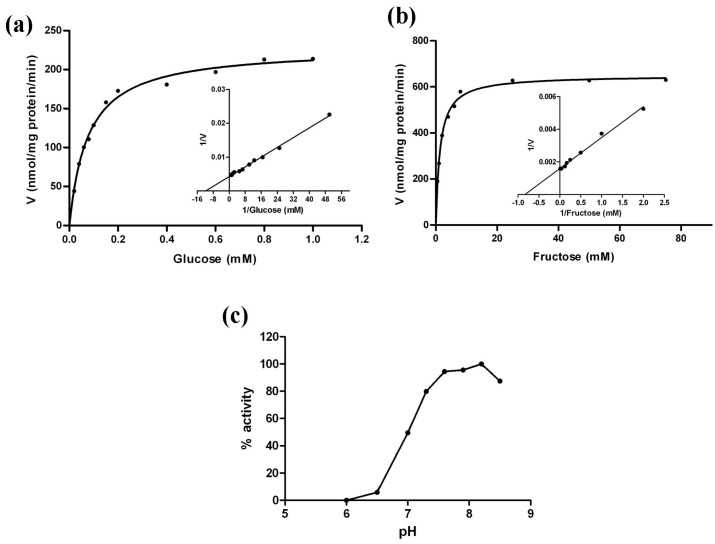
Enzyme activity analysis of MeHXK2 from yeast extracts (**a**) Hexose phosphorylation activity of MeHXK2 towards glucose; (**b**) Hexose phosphorylation activity of MeHXK2 towards fructose. Note the different scales of sugar concentrations; (**c**) Comparison of the pH and the hexose phosphorylation activity of MeHXK2. V: nmol of glucose 6-phosphate or fructose 6-phosphate that produce 1 mg of protein in 1 min.

To further investigate the enzymatic properties of MeHXK2, the crude proteins were extracted from cells of the yeast triple mutant YSH7.4-3C (*hxk1*, *hxk2*, *glk1*) transformed with pDR195-*MeHXK2* and pDR195, which were cultured in medium with galactose as the sole carbon source and lacking uracil. The results showed that the crude protein extracts from the YSH7.4-3C yeast cells transformed with pDR195 alone had no measurable hexose phosphorylation activity; while the crude protein extracts from the YSH7.4-3C yeast cells carrying pDR195-*MeHXK2* had activity towards glucose and fructose with *K*_m_ values of 78 µM for glucose and 1321 µM for fructose. Thus, the affinity of *MeHXK2* for glucose was higher than that for fructose (Figure 13a,b, Table 2). However, the maximal activity towards fructose was higher than that towards glucose. The optimum pH for the enzyme activity of MeHXK2 was ~8.2 (Figure 13c).

## 3. Discussion

### 3.1. Identification and Characterization of MeHXK Genes

HXKs play a pivotal regulatory role during cassava starch synthesis, during which HXK activity is involved in sucrose metabolism and regulates the expression of SBEs in the cassava tuber root [28]. However, no further information about the *HXK* gene family has been reported in cassava. In the present study, seven *HXK* genes (*MeHXK1–7*) were isolated from the cassava cultivar SC8. Based on the subcellular localization prediction and N-terminal sequence analysis of MeHXK1–7, they were divided into type A (MeHXK1) and type B (MeHXK2–7). Previous studies have reported that the members of the *HXK* gene family vary among plant species. In *A. thaliana*, there are six members [6]; in *Z. mays*, there are nine members [32]; and in *O. sativa*, there are ten members [18]. The seven *MeHXK* genes are distributed among seven chromosomes (Figure 1). However, nine *ZmHXK* genes, ten *OsHXK* genes, and six *AtHXK* genes are located on three chromosomes of *Z. mays*, *O. sativa*, and *A. thaliana*, respectively [6,18,31]. Most of the MeHXKs (MeHXK2–5) are encoded by nine exons, and the exon-intron structures are consistent with those of all the reported *HXK* genes in other plants [6,18,32,33]. Phylogenetic analysis of the 56 HXK proteins from eight plants showed that the MeHXKs are most closely related to the HXKs from *R. communis* and *P. trichocarpa* (Figure 4). These three species belong to the order Malpighiales.

An alignment analysis of amino acid sequences showed that MeHXK1–5 all had typical conserved regions, which were consistent with the reported HXKs in other plants [6,34]. Motif analysis showed that MeHXK1–5 have typical HXK motifs, which are the same as those in PtHXKs from *P. trichocarpa* and RcHXKs from *R. communis* (Figure 6). However, MeHXK6 has lost the C-terminal sequence and two conserved regions in comparison to the other MeHXKs; MeHXK7 has lost several conserved regions (phosphate 1, connect 1, and phosphate 2) (Figure 2). The lost conserved regions and motifs might affect the catalytic function of MeHXK6–7. MeHXK3–4 have an unusual insertion-deletion mutation at the N-terminal sequence containing the adenosine phosphate-binding region, which is different to that of the other MeHXK proteins. This phenomenon was also found in the HKL proteins such as AtHXK1–3 from *A. thaliana*, OsHXK3 and 10 from *O. sativa*, and PtHKL1–2 from *P. trichocarpa*. AtHKL1 lacks a catalytic function, and only has a regulatory function [35]. This result suggests that MeHXK3–4 might be HKL proteins.

The 3D structural models of MeHXK1–5 showed that the N-terminus sequences form a small domain and the C-terminus sequences form a large domain. These domains are separated by a deep cleft that contains many residues involved in the enzyme active site. This structure is typical of HXK proteins [29], implying that MeHXK1–5 have the same structure and catalytic function as the HXK proteins of other plants. MeHXK6 only has the N-terminal sequence and folds into a small domain, which implies that this protein might lack a catalytic function. Analysis of the theoretical position of the glucose-binding site in MeHXKs determined that MeHXK1–2 and 5 contain all the eight conserved catalytic residues, while MeHXK3–4 and 6 lack several catalytic residues. These differences in the presence of the catalytic residues might result in the different MeHXKs having varying physiological functions.

### 3.2. Differential Expression and Enzymatic Activity of MeHXK1–7

The tissue-specific expression pattern of *MeHXK1–7* could provide a basis for understanding their functions in cassava plant development. The expression pattern of *MeHXK1–7* in various organs or tissues was examined at the mature tuber stage. Our results found that *MeHXK1–6*, but not *MeHXK7*, were expressed in all the tested organs and tissues, and the expression of *MeHXK2* was highest among all the *MeHXKs*. *MeHXK1* showed vigorous activity in most of the tested organs and tissues, except tuber xylem, while *MeHXK4–5* were mainly active in flowers. The levels of *MeHXK6* varied, and the expression of *MeHXK3* was low in all the tested organs and tissues. This suggests that the roles of MeHXKs in different organs and tissues may vary. Similar results were found for other *HXK* genes. For instance, the expression of *AtHXK1* from *A. thaliana* was higher than other *AtHXKs* in all the examined organs (young leaves, mature leaves, root, flower, silique, and stem), which is a similar expression pattern to that of *MeHXK2*, and both of these genes were classified into group VI in the phylogenetic tree (Figure 5).

It was reported that HXKs play a pivotal role for cassava starch synthesis by regulating the expression of SBEs through sugar signaling in cassava tuber roots. To investigate the temporal expression of *MeHXK1–7* during cassava tuber root development, qPCR analysis was performed using tuber phloem and xylem at the initial tuber stage (90 days), the expanding tuber stage (135 and 180 days, the main period of starch accumulation), and the mature tuber stage (225 and 270 days). The results showed that *MeHXK2* was the most active gene among *MeHXK1–7* in cassava tuber tissues with high activity at the initial and expanding tuber stages and lower activity at the mature tuber stage. In addition, the enzymatic activity of HXK was higher at the initial and expanding tuber stages in tuber phloem and xylem, which was consistent with the results for the *HXK* gene expression during cassava tuber root development (Figure 11). It was reported that HXK is a crucial component in the sugar signaling network for regulating the expression of genes related to cell division and sucrose metabolism in the roots of *Vicia faba* [20]. The results from the gene expression and enzymatic activity analyses suggest that *MeHXK2* might the key gene of hexose phosphorylation during cassava tuber root development, which is involved in sucrose metabolism to regulate the accumulation of starch.

Further investigation showed that *MeHXK2* could complement a HXK-deficient yeast strain to grow on medium with glucose or fructose as the sole carbon source, indicating that MeHXK2 can catalyze the phosphorylation of hexose. The affinity of plant HXKs for glucose is much higher than its affinity for fructose [9]. The LeHXK1 from *L. esculentum* has much lower *K*_m_ value for glucose (77 µM) than fructose (10,560 µM), and its *V*_max_ value for fructose (282.7 nmol/mg·pr/min) is higher than glucose (195.7 nmol/mg·pr/min) [36]. Similarly, the *K*_m_ value of MeHXK2 for glucose (78 µM) is lower than fructose (1321 µM), and its *V*_max_ value for fructose (664.0 nmol/mg·pr/min) is higher than glucose (228.1 nmol/mg·pr/min).Thus, MeHXK2 is characterized as a hexokinase with preferential affinity to glucose.

## 4. Materials and Methods

### 4.1. Plant Materials

The plant materials were collected from cassava (*M. esculenta* Crantz) variety SC8 plants (provided by the Tropical Crops Genetic Resources Institute, Danzhou, China), which were normally planted in the field and used in all the experiments. Cassava leaves were used for *HXK* genes cloning. Cassava leaves, stems, fruits, tuber phloems, and tuber xylems were harvested at 225 days, and male and female flowers were harvested at 200 days after planting for differential gene expression analysis. Samples of tuber phloem and xylem were collected at the initial tuber stage (90 days), the expanding tuber stage (135 and 180 days), and the mature tuber stage (225 and 270 days) for the differential expression analysis of *MeHXK* genes in sink organs during tuber root development. Three biological samples (leaf, stem, tuber phloem, tuber xylem, male flower, female flower, fruits from different plants) were collected for each analysis. All fresh materials were preserved at −80 °C after freezing in liquid nitrogen for subsequent RNA isolation.

### 4.2. Cloning of Full-Length MeHXKs

To identify all the *MeHXK* genes in the cassava genome, a systematic BLAST analysis of the cassava genome database (http://www.phytozome.net/cassava) [37] was carried out using the published sequences of the *HXK* genes in *R*. *communis* (*RcHXK1–3* and *RcHKL1–2*) and *A*. *thaliana* (*AtHXK1–3* and *AtHKL1–3*). Full-length cDNAs of the *MeHXK* genes were isolated by reverse transcription PCR using gene-specific primers (Table 3). Total RNA was isolated from the leaf samples using RNAplant Plus reagent (TianGen, Beijing, China). First-strand cDNA was synthesized from 1 μg of each total RNA sample using random primers and the RNA PCR Kit (AMV) Ver. 3.0 (TaKaRa Biotechnology, Otsu, Japan), following the manufacturer’s instructions. Each PCR fragment was separately cloned into the pMD18-T vector (TaKaRa Biotechnology) and the sequencing of independent clones was performed on both strands by Sangon Biological Engineering Technology and Services (Shanghai, China).

### 4.3. Sequence Analysis of MeHXKs

The deduced amino acids of *MeHXK1–7* were analyzed by multiple alignment using DNAman 6.0 software (Lynnon Biosoft, Quebec City, QC, Canada). Chromosomal locations and gene structures were researched for all *MeHXK* genes obtained from the cassava genome database (http://www.phytozome.net/cassava). The gene schematic structures were drawn by the Gene Structure Display Server (http://gsds.cbi.pku.edu.cn/index.php). Comparison of the N-terminal regions was performed using the subcellular prediction localization program TargetP 1.1 (http://www.cbs.dtu.dk/services/TargetP/) and prediction of the membrane anchored segment was done using TopPred2 (http://www.sbc.su.se/~erikw/toppred2/) [38]. For the phylogenetic analysis, *HXKs* from *P. patens*, *A. thaliana*, *O. sativa*, *N. tabacum*, *R. communis*, *S. lycopersicum*, and *P. trichocarpa* were aligned using the Muscle software, and the phylogenetic tree was constructed by the neighbor-joining method with 1000 bootstrap resamplings using MEGA7 software [31]. The conserved motifs of HXK proteins from *M. esculenta*, *P. trichocarpa*, and *R. communis* were predicted using the MEME web server.

### 4.4. Homology Modeling of MeHXKs

3D structural models of MeHXKs were generated using the homology modeling server SwissModel [39] using the HXK protein crystal structure complexed with glucose from *S. mansoni* (Protein Databank ID 1BDG) as a template. To predict the theoretical position of glucose binding with MeHXKs, the 3D structural models of MeHXKs were further structurally aligned with 1BDG in complex with glucose using PyMOL (http://pymol.sourceforge.net/). The predicted catalytic residues of MeHXKs were also displayed using PyMOL.

### 4.5. qPCR Analysis

Total RNAs were extracted from each biological sample (leaf, stem, tuber phloem, tuber xylem, male flower, female flower, fruits from three different plants) using RNAplant Plus reagent (TianGen, Beijing, China) according to the manufacturer’s protocol. Two micrograms of each RNA sample was used as the template for first-strand cDNA synthesis using a PrimeScript™ RT reagent Kit with gDNA Eraser (TaKaRa Biotechnology), according to the manufacturer’s protocol. qPCR was performed using gene-specific primers (Table 4). A housekeeping gene, *β-tubulin*, was amplified as an internal control [26]. qPCR was performed on an ABI 7900Fast instrument (Applied Biosystems, Foster City, CA, USA) using SYBR^®^ Premix Ex Taq™ II (Tli RNaseH Plus) (TaKaRa Biotechnology), cycling between 95 and 60 °C, as per the manufacturer’s protocol, for 45 cycles. The threshold cycle (*C*_t_) was determined using proprietary software from Applied Biosystems. The relative expression data was analyzed using the 2^−ΔΔ*C*t^ method [40]. Three technical replicates were analyzed for each biological sample.

### 4.6. Activity Analysis of HXKs

HXK activity in tuber phloem and tuber xylem at different tuber developmental stages were measured using a HXK assay kit (Solarbio Science and Technology, Beijing, China), according to the manufacturer’s protocol. HXK activity was measured as the total glucose-phosphorylating capacity and expressed in U/g fresh weight. Three technical replicates were analyzed for each biological sample (tuber phloem and tuber xylem from three different plants).

### 4.7. Yeast Complementation and Enzymatic Analysis of MeHXK2

A HXK-deficient yeast strain (YSH7.4-3C) with triple knockouts of *HXK1*, *HXK2*, and *GLK1* in the W303-1A background was kindly provided by Stefan Hohmann [20]. The yeast shuttle vector pDR195, containing *URA3* as a selective marker, was used for transformation. The cDNA of *MeHXK2* was inserted into the XhoI/BamHI sites within pDR195, and the new plasmid was verified by sequencing and designated as pDR195-MeHXK2. YSH7.4-3C yeast cells were grown on YPgal medium consisting of 1% yeast extract, 2% Bacto™ peptone, and 2% galactose. The selective media for the transformed colonies contained 0.67% yeast nitrogen base, 2% of a carbon source (d-glucose or d-fructose), some appropriate amino acids, and no uracil. As a control, the YSH7.4-3C yeast cells were transformed with the pDR195 vector alone.

YSH7.4-3C yeast cells, transformed with either pDR195 or pDR195–MeHXK2, were grown in 30 mL of −URA/galactose liquid media for 3 days. Yeast cells were spun down for 5 min at 7000× *g* and yeast protein was extracted using a yeast total protein extraction kit (Sangon, Shanghai, China) following the manufacturer’s instructions. Total protein concentrations were determined using a Bradford protein assay kit (Sangon) with bovine serum albumin as the protein standard. Total protein extracts were used as crude enzyme extracts for subsequent enzymatic analysis. Determination of *K*_m_ values for fructose and glucose and the pH-dependence of the hexose phosphorylation activity of MeHXK2 was performed using a HXK assay kit (Solarbio Science and Technology).

## 5. Conclusions

In this study, seven *HXK* genes (*MeHXK1–7*) were isolated and identified. All MeHXKs were divided into type A (MeHXK1) and type B (MeHXK2–7). MeHXK1–5 all had typical conserved regions and similar protein structures to the HXKs in other plants; while MeHXK6–7 lacked some of the conserved regions. Among *MeHXKs*, *MeHXK2* was the most active gene in all the tested organs or tissues (leaves, stems, fruits, tuber phloem, tuber xylem, male and female flowers) and in cassava tuber root development. MeHXK2 showed activity towards glucose and fructose, with a higher affinity for glucose than fructose. These results suggest that *MeHXK2* might a key gene for hexose phosphorylation in cassava plants during tuber root development.

## Figures and Tables

**Table 1:** Basic information of the seven cassava hexokinase genes (*MeHXKs*).

Gene Name	Accession Number	Open Reading Frame (ORF) Lengths (bp)	Protein Lengths (a.a)	pI	*M*w (Da)
*MeHXK1*	KJ417433	1488	496	6.20	53,356.2
*MeHXK2*	KJ417434	1494	498	6.01	53,901.4
*MeHXK3*	KJ417435	1524	508	6.91	55,303.8
*MeHXK4*	KJ417436	1491	497	7.70	54,364.9
*MeHXK5*	KJ417437	1482	494	5.81	53,440.8
*MeHXK6*	KJ417438	861	287	7.05	30,714.7
*MeHXK7*	KJ417439	933	311	6.43	33,671.1

**Table 2:** Kinetic parameters of the MeHXK2 enzyme expressed in yeast.

Substrate	*K*_m_ (mM)	*V*_max_ (nmol/mg·pr/min)	*K*_m_/*V*_max_
Glucose	0.078	228.1	33.59
Fructose	1.321	644.0	2.05

**Table 3:** Gene specific primers of *MeHXKs* used for RT-PCR amplification.

Gene	Forward Primer (5′ to 3′)	Reverse Primer (5′ to 3′)
*MeHXK1*	TTTCACGTTCTTTGATCTCACCG	TTAGCCAATGAATCCCAGTTGC
*MeHXK2*	GCAGCTAGTGGTCATGGGAAAGG	TTTCACATACCCAATTTCGAGTTAGC
*MeHXK3*	GAAACGGCGACGTTATGGTTGA	ATTTCATCTTTCCAGGTGGGTT
*MeHXK4*	GTAGTGCTGATGGGAAGGGTGA	TGGATAGCGTACAGTAGCTATAAA
*MeHXK5*	TATCACAATCAACCTTCTTTCTTCA	ATTCGCATTATCGTTAAGTTGAGG
*MeHXK6*	GTACTCATGGGAAAGGTGGCG	ATAAGCCGAATGAAAAAGGATGAC
*MeHXK7*	CTCTTGTTGTAGTGAAGGTTTTGA	ATCAGTTTACTCATTAGGAATCAGG

**Table 4:** Primers for *MeHXKs* used for qRT-PCR amplification.

Gene	Forward Primer (5′ to 3′)	Reverse Primer (5′ to 3′)
*MeHXK1*	CATTGGACGGAGGGCTGTTT	GTCATCCTTTTTCATTCGGCTTAT
*MeHXK2*	ATGGTCTTCTTCCTAAATCTGGG	GTGGAACAACATCACCAAAGAAG
*MeHXK3*	GCGACGTTATGGTTGAGGTTAT	ACTCTTCCAACTCCCTCAACAC
*MeHXK4*	GCGGTGGCGATGAGTGTC	CTCCAACTCCCTCAATAATCCC
*MeHXK5*	AGTATGTGCTACCGCTGTGGC	CCCACTTATCACCCGTCCTCA
*MeHXK6*	TCTAACATCGTCTCGGTGGCT	ACAATCCTTTCTCGTTCCCGC
*MeHXK7*	TTACTTCACTGCCCAGCGGACTT	TCTTGCGGTTGCTTCACCATCAT
